# Rates of Textbook Outcome Achieved in Patients Undergoing Liver and Pancreatic Surgery

**DOI:** 10.3390/jcm13216413

**Published:** 2024-10-26

**Authors:** Celia Villodre, Candido F. Alcázar-López, Silvia Carbonell-Morote, Paola Melgar, Mariano Franco-Campello, Juan Jesus Rubio-García, José M. Ramia

**Affiliations:** 1 Department of Surgery, General University Hospital Dr. Balmis, 03010 Alicante, Spain; candidoalcazar@hotmail.com (C.F.A.-L.); sayasilv@icloud.com (S.C.-M.); paomelgar@hotmail.com (P.M.); marianofranco@hotmail.com (M.F.-C.); juanjesus1010@gmail.com (J.J.R.-G.); jose_ramia@hotmail.com (J.M.R.); 2 Institute of Health and Biomedical Research of Alicante (ISABIAL), 03010 Alicante, Spain; 3 Faculty of Medicine, Miguel Hernández University, 03202 Elche, Spain

**Keywords:** textbook outcomes, liver surgery, pancreatic surgery, benchmarking

## Abstract

**Backgorund:** Textbook outcome (TO) is a composite measure that reflects the most desirable surgical results as a single indicator. The aim of this study was to assess the achievement of TO at a hepatopancreatobiliary (HPB) surgery unit in a Spanish tertiary hospital. **Methods:** We performed a retrospective observational study of all consecutive patients who underwent HPB surgery over a 4-year period. Morbidity according to the Clavien-Dindo classification at 30 days, hospital stay, risk of morbidity and mortality according to the POSSUM, and mortality and readmissions at 90 days were recorded. TO was considered when a patient presented no major complications (≥IIIA), no mortality, no readmission, and no prolonged length of stay (≤75th). **Results:** 283 patients were included. Morbidity >IIIA was reported in 21.6%, and 5.7% died; the median postoperative stay was 4 days. TO was achieved in 56.2% of patients. Comparing patients who presented TO with those who did not, significant differences were recorded for the type of procedure and the expected risk of morbidity and mortality calculated according to the POSSUM scale. There were significant differences between patients with major resections (TO rates: major hepatectomy (46.3%) and major pancreatectomy (52.5%)) and those with minor resections (TO rates minor hepatectomy (67.7%) and minor pancreatectomy (40.4%)). **Conclusions:** TO is a useful management tool for assessing postoperative results.

## 1. Introduction

Postoperative morbidity and mortality are major public health concerns. Avoidable postoperative complications result in unnecessary patient suffering, prolonged hospital stay, and misuse of hospital resources [1]. Liver surgery is a major procedure with morbidity rates ranging from 17% in benign cases to 27% in malignant pathology, with a risk of mortality of up to 5% [2]. Pancreatic surgery is another complex intervention due to a combination of the patients’ characteristics and the procedures involved. Hepatic, biliary, and pancreatic (HPB) surgery has evolved significantly in recent decades, and performing pancreatic and liver procedures at high-volume centers has significantly reduced rates of adverse outcomes [3]. In fact, perioperative mortality has fallen notably in specialized centers, with mortality rates of only 1% to 2% after resection of the pancreas [4] and 3% to 5% in resections of the liver [5].

The “textbook outcome” (TO) is a composite measure that reflects the most desirable surgical outcomes as a single indicator. It was originally described by a group of colorectal surgeons in the Netherlands in order to quantify hospital activity on the basis of the results obtained [6]. A TO is achieved when all the desirable results are obtained after surgery, all the proposed measures are successfully applied, and the hospital stay is optimal. The six parameters included in this first assessment of TO were survival at 30 days, radical resection, no reintervention, no ostomy, no complications during the first 30 days, and a postoperative stay of 14 days maximum [6].

Since its design, the concept of TO has been evaluated in several surgical subspecialties, such as esophagogastric oncological surgery [7] and peritoneal carcinomatosis surgery [8], in attempts to improve the quality of care. In their definition of TO in liver and pancreas surgery, Merath et al. [9] proposed four requirements: no postoperative complications at 30 days, no readmission in the first 90 days, no mortality at 90 days, and no prolonged postoperative stay. The aim of the present study is to assess the achievement of TO at an HPB unit in a Spanish tertiary care hospital.

## 2. Methods and Materials

This retrospective observational study included all patients undergoing consecutive HPB surgery between January 2017 and December 2020. The inclusion criteria were age over 18 and prior elective surgery with any of the following procedures: minor pancreatic resection (i.e., distal pancreatectomy [DP] or other partial pancreatectomies, enucleation of pancreatic tumor), major pancreatic resection (i.e., cephalic pancreatoduodenectomy [PD] or total pancreatectomy), complex biliary surgery (radical resection of the main bile duct with or without hepatectomy), minor hepatic resection (<3 segments, metastasectomies, segmentectomies), and major hepatic resection (>3 segments resection and right and left hepatectomy). The following procedures performed at the unit during the study period were not included in the study: inferior vena cava resection surgery, laparoscopic cholecystectomy, liver transplantation, and non-HPB surgery (i.e., retroperitoneal tumors).

The study was conducted in accordance with the Declaration of Helsinki (2013) and was approved by the Institutional Ethics Committee of the Hospital General Universitario Dr. Balmis (Alicante) (Ref CEIm PI2023-034). The need to obtain patients’ informed consent was waived since the study was retrospective and observational and entailed no risk. The work has been reported in line with the STROCSS criteria [10].

Demographic variables such as age and sex were recorded, as were diagnosis, surgical procedure performed, and postoperative data (30-day morbidity according to the Clavien-Dindo classification [11], mortality at 90 days, hospital stay, and readmissions at 90 days). At our unit, we apply the ERAS protocol (Enhanced Recovery After Surgery) [12] in all patients.

The analysis also included the physiological and operative variables in the POSSUM system (POSSUM: Physiological and Operative Severity Score for the enUmeration of Mortality and morbidity) [13], as well as the expected risk of morbidity and mortality according to this scale. The data were obtained from a database in Access 2003^®^ format (Microsoft Corporation, Redmont, Washington, DC, USA), hosted on the hospital’s internal network, from which anonymized data tables were generated for statistical analysis. This network prospectively has recorded the variables of the POSSUM scale for all emergency and scheduled surgical interventions performed at the General Surgery service since 2009 [14,15].

### 2.1. Definitions

Patients who met the following four parameters were considered to present TO: no major postoperative surgical complications (no complications with Clavien-Dindo grade III or higher), no prolonged length of hospital stay (LOS), survival at 90 days, and no readmission (to any hospital) at 90 days after discharge [9]. A prolonged LOS was defined as LOS during the index hospitalization >75th percentile for each procedure.

### 2.2. Statistical Analysis

Quantitative data were expressed as medians and interquartile range (IQR) or means and standard deviation (SD), and qualitative data were expressed as frequencies or percentages. Differences between groups in the case of quantitative variables were analyzed using the non-parametric Mann–Whitney U test, and differences between percentages or frequencies using Pearson’s Chi-square test.

Moreover, we used a chi-square automatic interaction detection (CHAID) decision tree model [16] described elsewhere to identify the best cutoff values for continuous variables.

Univariate and multivariate logistic regression analyses were performed to investigate possible relationships between the characteristics of the variables and the achievement of TO.

Calculations were performed using the programs Microsoft^®^ Excel for Mac, version 16.49 and SPSS^®^ for Mac, version 26.0 (SPSS Inc., Chicago, IL, USA). A value of *p* < 0.05 was considered statistically significant.

## 3. Results

A total of 283 patients who underwent HPB surgery were included. The median age was 65 years (57–72), with a preponderance of men (65.4%) (Table 1). The procedures performed were major hepatectomies 14.5%, minor hepatectomies 45.9%, PD 21.6%, DP 9.5%, and complex biliary surgery 8.5%.

TO was achieved in 56.2% of the patients. In all, 21.6% presented major complications (Clavien-Dindo ≥ IIIA); mortality was 5.7%; the median postoperative stay for each procedure was 5 days (3–6) for minor pancreatic resection, 8 days (6–16) for major pancreatic resection, 3 days (2–4) for minor hepatic resection, 4 days (3–8) for major hepatic resection, and 9 days (3–21) for complex biliary surgery. The readmission rate was 20.8%. The expected mean morbidity risk, according to the POSSUM scale, was 38.4%, and the risk of mortality was 9% (Table 1).

Comparison of the patients with TO and those without revealed significant differences according to the type of procedure (*p* < 0.001), the POSSUM physiological score (*p* = 0.014) and surgical variables (*p* = 0.045), the expected risk of morbidity (*p* < 0.001) and mortality (*p* < 0.001) calculated according to the POSSUM scale (Table 1). Patient’s age and gender were not significantly associated with the achievement of TO.

**Figure 1:**
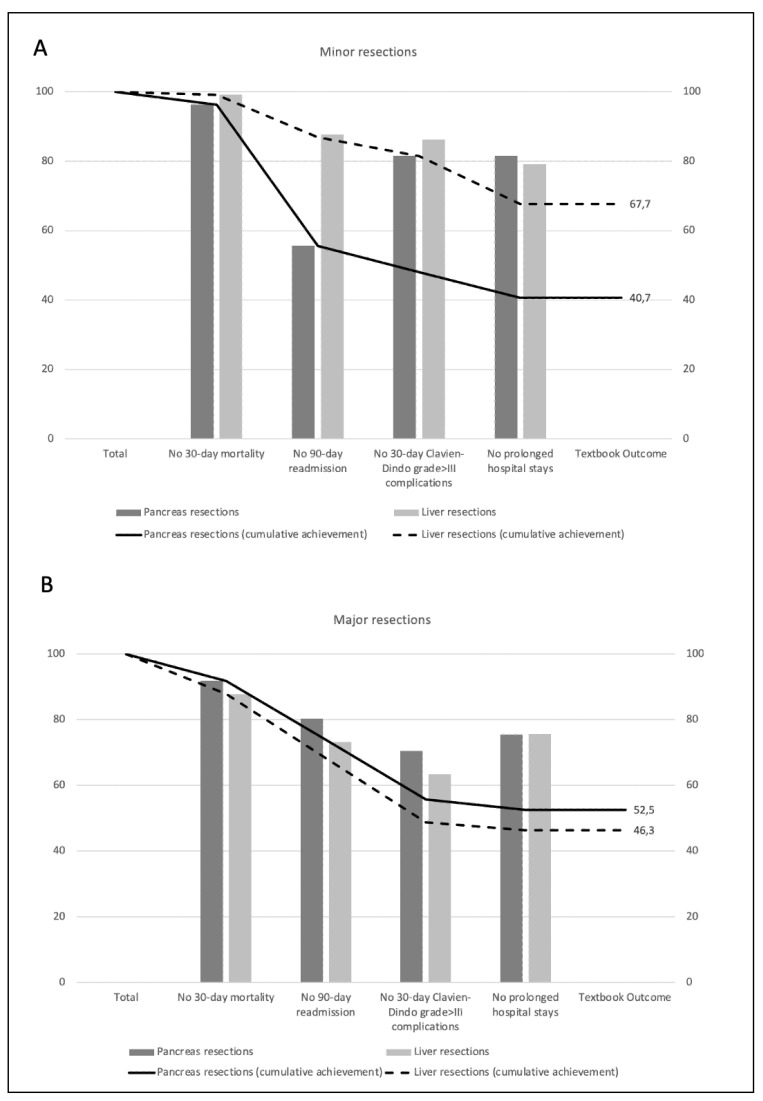
(**A**). Distribution of TO according to its definition in patients undergoing minor liver and pancreas resections. (**B**). Distribution of TO according to its definition in patients undergoing major liver and pancreas resections.

There were significant differences in the achievement of TO between patients undergoing major resections (major hepatectomy 46.3%, major pancreatectomy 52.5%) and those undergoing minor resections (minor hepatectomy 67.7%, minor pancreatectomy 40.7%), both in terms of overall rates of TO and in terms of the individual variables that comprise it (Table 2, Figure 1).

We present the prevalence of events that caused the failure to achieve TO (Figure 1 and Table 3). Overall, the most likely event to lead to failure to achieve TO was a major complication followed by prolonged LOS. However, in minor pancreatic resection and complex biliary resection, readmission within 90 days influences more (n = 12, 75% and 8, 53.3%, respectively). In addition, a comparison of all the variables on the POSSUM scale revealed significant differences only in intraoperative blood loss (Table 4).

**Figure 2:**
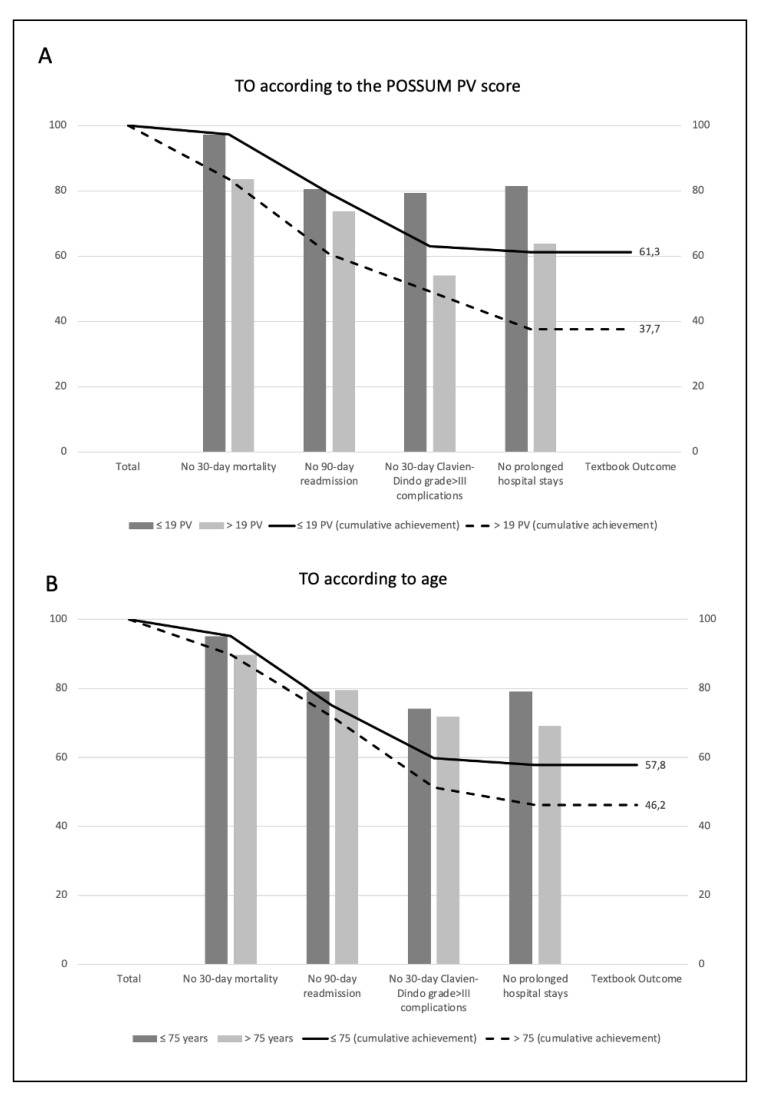
(**A**): Distribution of results according to their definition in patients according to age, taking 75 years as the cut-off point. (**B**): Distribution of results according to their definition in patients based on the POSSUM PV score.

A CHAID decision tree model strategy was used to identify the best cut-off value for age and POSSUM physiological score associated with achievement of TO. The best fit for the CHAID decision tree model to the POSSUM physiological score was 19 points (Pearson’s Chi-square: 10.8; *p* = 0.008). Taking this value as an indicator of the patient’s preoperative status, we found significant differences (*p* < 0.001) in the overall rates of TO achieved and in its components assessed individually (Figure 2A). No best association was identified between age and achieving TO. Classifying patients taking 75 years as the cut-off point (P75 of the population), no differences were observed in the distribution of the components of TO or in the achievement of TO (*p* = 0.174) (Figure 2B).

Univariate logistic regression analysis suggested that the variables hemoglobin, type of procedure, and POSSUM physiological score might be significant predictive factors for the achievement of TO (Table 5). Specifically, hemoglobin below 10 g/dL or greater than 18 g/dL was associated with a 69% decrease in the chance of achieving a TO (OR 0.31, 95% CI 0.11–0.87), *p* = 0.026). In addition, patients with a POSSUM physiological score ≤19 had a 62% decrease in the probability of achieving a TO (OR 0.38, 95% CI 0.54–0.66, *p* < 0.001), while patients with a comorbidity score of >5 had a 58% lower chance of a TO (OR 0.42, 95% CI 0.21–0.69, *p* < 0.001). Compared with patients who underwent minor liver resection, patients who underwent minor pancreatic procedures had a 67% lower chance of having a TO (OR 0.33, 95% CI 0.14–0.77, *p* = 0.01). Similarly, patients undergoing a major liver resection had a 59% (OR 0.41, 95% CI 0.2–0.84, *p* = 0.015) lower chance of a TO, and patients who had a major pancreatic resection had 47% (OR 0.53, 95% CI 0.28–0.98, *p* = 0.044). However, in the multivariate regression analysis with these three parameters, only the type of procedure and POSSUM physiological score remained statistically significant (Table 5).

## 4. Discussion

This study has confirmed the usefulness of the information obtained by combining the sum of several expected outcome parameters into a single outcome measure like TO. The rates of TO obtained in our series (56.2%) are similar to those published internationally [9]. The percentage of TO according to procedures varies from 37.5% of complex biliary surgery to 67.7% of minor liver resections, which represent 45.9% of the total patients included in the analysis. As expected, the percentage of patients with TO was higher for minor liver surgeries, 67.7% (46.3% for major hepatic surgery), but not in minor pancreatic surgery, where 40.7% of patients achieve TO, lower than 52.5% in major pancreatic resections. The parameters that most influenced the failure to achieve TO in minor pancreatic surgery were equally readmission (44.4% patients readmitted) and complications (44.4% patients with major complications), while in major pancreatic surgery, these parameters were complications (62.1% patients). These results suggest that our patients who underwent minor pancreatic resection had a shorter postoperative stay but required more readmissions.

The parameters that comprise TO—complications, readmissions, prolonged postoperative stay, and mortality—are frequently used as markers of the quality of care in surgery [4]. Most readmissions after pancreatic surgery are associated with the procedure and occur within the first 30 days [17]. Readmission after liver resection is recorded in approximately one in seven patients [18]. Patients who presented a postoperative complication are more than five times more likely to be readmitted. A total of 59 of 283 patients (20.8%) needed a readmission. Major procedures had a higher readmission rate, 26.8% for liver resections and 19.7% for pancreatic resections.

Merath et al. [9] highlighted the negative effect of prolonged hospital stay on the achievement of TO. In our study, this value emerges as the second most influential, it only has a higher rate in minor liver resections. Overall, our postoperative stay was short. As regards major surgery, hospital stay was not prolonged in 75.6% of patients undergoing major liver surgery (LOS ≤ 16 days) and in 75.6% of those undergoing major pancreatic surgery (LOS ≤ 8 days). In minor procedures, 81.5% of minor pancreatic resections and 79.2% of minor liver resections did not require a prolonged stay.

Patients’ physiological status and the difficulty of the intervention had a stronger effect on the achievement of TO than their age. Patients aged over 75 who required major surgery but had good physiological status obtained TO results like the rest. A careful assessment of preoperative frailty and personalized decision-making is necessary for elderly patients who require HPB surgery.

Preoperative anemia was one of the predictive factors of the failure to obtain TO. A preoperative hemoglobin level below 10 g/dL or above 18 g/dL predicts that these patients are 73% less likely to achieve TO. In our study, 6.4% of the patients operated on belonged to this group, and 12 of them (66.7%) did not obtain TO. The increasing burden of chronic diseases (heart disease, kidney disease, cancer) coupled with an aging population makes it difficult to optimize preoperative anemia [19]. It is well known that both anemia and the perioperative blood transfusions it entails pose a significant threat to postoperative rehabilitation and increase the risk of poor outcomes [20]. Despite advances in hemorrhage control in surgery, it is essential to optimize the red blood cell mass to reduce the need for perioperative transfusions. Prior to major elective surgery, patients with anemia should be identified and treated using protocols (i.e., search for a reversible cause, management of iron deficiency, transfusion if necessary) [19]. Type of procedure was another negative predictor for TO compared with minor liver resection; the study confirmed that TO was less likely in the setting of major surgery.

In a recent systematic review, Pretzsch et al. [21] highlight TO is a multidimensional measure reflecting the ideal outcome after surgery but is still heterogeneously defined. Ideally, included parameters should be disease- and surgery-specific. A current definition of TO in HPB surgery should include the terms ‘no prolonged LOS’, ‘no complications’, ‘no readmission’, and ‘no deaths’, like our definition. The median rate of TO achievement was 38 percent, ranging widely from 15 to 70 percent. Major obstacles in achieving TO were ‘complications’, ‘prolonged LOS’, and ‘readmission’. This review included heterogeneity of definitions of TO that impeded fair comparisons of TO rates between institutions, so meta-analysis was inappropriate.

Recently, equivalent rates of achievement of TO have been reported in patients undergoing minimally invasive pancreaticoduodenectomy and in patients undergoing open pancreaticoduodenectomy after performing propensity score matching [22]. In the current study, the use of open or laparoscopic approaches was not compared since laparoscopic surgery was not widely used during part of the study period; over the years, however, its use has progressively increased. The comparison of open and laparoscopic approaches might represent an interesting future line of research; although no differences have been demonstrated so far, future studies may find that laparoscopic surgery achieves improved results. Likewise, Endo Y et al. recently published that patients with HCC undergoing open or minimally invasive resection had a comparable percentage of TO, although the minimally invasive approach was associated with a short hospital stay [23].

To define TO in liver surgery (TOLS), in minimally invasive and open resection, an international expert Delphi consensus was carried out using a modified Delphi method [24]. The TOLS definition included the absence of intraoperative grade ≥ 2 incidents, postoperative bile leakage grade B/C, postoperative liver failure grade B/C, 90-day major postoperative complications, 90-day readmission due to surgery-related major complications, 90-day/in-hospital mortality, and the presence of R0 resection margin. This consensus is after our analysis, they did not include prolonged LOS and added four variables; however, except for the presence of R0 resection margin and the absence of intraoperative grade ≥2 incidents, the other two are also mostly classified as major complications.

Several limitations of the study should be considered when interpreting the results. Due to the retrospective design, there may have been a selection bias and pitfalls in the database that we have tried to check deeply. Also, the study used 30-day rather than 90-day complication rates. Although using the 30-day limit may have underestimated the total incidence of complications, these data are recorded prospectively at our department, thus reducing the probability of bias. What is more, the use of the POSSUM scale and the analysis of all its variables over a period of more than ten years enhances the value of the study since it allows us to assess the TO composite measure with a tool that has been used to audit results at our service for an extended period. We have measured TO as in previous manuscripts related to techniques (major/minor hepatectomies and PD/DP). We think that possible bias related to stage, native disease, or neoadjuvant therapy could marginally exist but has not been included in the measurements of TO before.

## 5. Conclusions

TO is a composite helpful measure to carry out healthcare quality improvement programs and compare hospital results. Multicenter studies in specific countries should be performed to define national TO cut-offs adapted to each health system. TO is a management tool for the assessment of postoperative results, provides information on surgical quality for the patient, and assists preoperative patient selection, with many advantages: measuring is not difficult to measure because the parameters included in the database are usually in the hospital managing database, so surgeons could be checked periodically simply by quickly asking managers for these data, and these data are easily understood and comparable with other HPB units.

## Figures and Tables

**Table 1:** Characteristics of patients with and without textbook outcome.

	Total	Textbook Outcome		
Characteristics	N = 283	No (n = 124)	Yes (n = 159)	*p* Value
Age (median, IQR)	65 (57–72)	67(58, 72)	64 (56, 72)	0.086
Gender %				
Male	65.4	62.9	67.3	0.441
Female	34.6	37.1	32.7	
POSSUM PS (median, IQR)	16 (13–19)	17 (14–21)	16 (13–18)	0.014
POSSUM OS (median, IQR)	14 (11–16)	15 (11–17)	14 (11–16)	0.045
ER Morbidity POSSUM (median + SD)	38.4 ± 20	43.7 ± 22.7	34.2 ± 16.6	<0.001
ER Mortality POSSUM (median + SD)	9 ± 8	11.3 ± 10.3	7.1 ± 5.1	<0.001
Procedure n, (%)				
Complex biliary surgery	24 (8.5)	15 (12.1)	9 (5.7)	
Major hepatectomy	41 (14.5)	22 (17.7)	19 (11.9)	
Minor hepatectomy	130 (45.9)	42 (33.9)	88 (55.3)	0.005
Major pancreatectomy	61 (21.6)	29 (23.4)	32 (20.1)	
Minor pancreatectomy	27 (9.5)	16 (12.9)	11 (6.9)	

POSSUM: Physiological and Operative Severity Score for the enUmeration of Mortality and morbidity; PS: physiological score; OS: operative score; ER: expected risk. *p* Value in italics when results were statistically significant (<0.05)

**Table 2:** Percentage of textbook outcome according to procedure.

Textbook Outcome	Minor Pancreatic Resection	Major Pancreatic Resection	Minor Hepatic Resection	Major Hepatic Resection	Complex Biliary Surgery
No	16 (59.3)	29 (47.5)	42 (32.3)	22 (53.7)	15 (62.5)
Yes	11 (40.7)	32 (52.5)	88 (67.7)	19 (46.3)	9 (37.5)
Total	27	61	130	41	24

**Table 3:** Events triggering failure to achieve TO.

Procedures						
	Total	Minor Pancreatic Resection	Minor Liver Resection	Major Pancreatic Resection	Major Liver Resection	Complex Biliary Resection
N (%)	124	16	42	29	22	15
Prolonged LOS	63 (50.8)	5 (31.3)	27 (64.3)	15 (51.7)	10 (45.5)	6 (40)
90-day readmission	59 (47.6)	12 (75)	16 (38.1)	12 (41.4)	11 (50)	8 (53.3)
30-day Clavien-Dindo grade>III complications	74 (59.7)	12 (75)	18 (42.9)	18 (62.1)	15 (68.2)	11 (73.3)
90-day mortality	16 (12.9)	1 (6.3)	1 (2.4)	5 (17.2)	5 (22.7)	4 (26.7)

**Table 4:** Distribution of POSSUM variables in patients with and without textbook outcome.

		Textbook Outcome		
		No (n = 124)	Yes (n = 159)	*p*
**Physiological variables**				
Age (years)	≤60	31.5	40.9	0.264
	61–70	33.1	28.3	
	≥70	35.5	30.8	
Cardiac signs	No failure	43.5	45.3	0.288
	Diuretic, digoxin, antianginal, or hypertensive therapy	46	49.1	
	Peripheral edema, warfarin therapy, borderline cardiomegaly	8.9	5.7	
	High jugular venous pressure, cardiomegaly	1.6	0	
Respiratory history	No dyspnea	73.4	77.4	0.630
	Dyspnea on exertion, minimal COPD * on chest X-ray	21.8	18.9	
	Limiting dyspnea (one flight), moderate COPD on chest X-ray	4.8	3.1	
	Dyspnea at rest (≥30/min), fibrosis or consolidation on chest X-ray	0	1	
Systolic blood pressure	110–129	71	65.4	0.288
(mmHg)	130–170 or 100–109	28.2	34.6	
	>170 or 90–99	0.8	0	
Pulse (beats/min)	50–80	95.2	95.6	0.862
	81–100 or 40–49	4.8	4.4	
Glasgow Scale	15	100	100	
Urea (mmol/L)	<7.5	86.3	91.8	0.814
	7.5–10	5.6	4.4	
	10.1–15	5.6	2.5	
	>15	2.4	1.3	
Sodium (mmol/L)	≥136	89.5	91.2	0.547
	131–135	10.5	8.2	
	126–130	0	0.6	
Potassium (mmol/L)	3.5–5	92.7	95	0.196
	3.1–3.4 or 5.1–5.3	5.6	3.8	
	2.9–3.1 or 5.4–5.9	0	1.3	
	<2.9 or >5.9	1.6	0	
Haemoglobin (g/dL)	13–16	47.6	60.1	0.087
	11.5–12.9 or 16.1–17	29.8	25.3	
	10–11.4 or 17.1–18	12.9	10.8	
	<10 or >18	9.7	3.8	
Leukocytes (×10^3^)	>4	83.9	87.4	0.580
	10.1–20 or 3.1–3.9	14.5	11.9	
	>20 or <3.1	1.6	0.6	
ECG	Normal	89.5	95	0.101
	Controlled atrial fibrillation at 60–90/min	6.5	4.4	
	Any other arrhythmia, 5 or more ventricular extrasystoles/min, Q waves, or changes in the S-T segment or in the T wave	4	0.6	
**Surgical variables**				
Surgical complexity	Major	61.2	69.8	0.133
	Major +	38.7	30.2	
Nº of interventions	1	100	100	
Blood loss (mL)	≤100	50	60.4	0.005
	101–500	38.7	36.5	
	501–1000	6.5	3.1	
	>1000	4.8	0	
Peritoneal soiling	No	95.2	98.1	0.372
	Minor (serous fluid)	3.2	1.9	
	Local pus	0.8	0	
	Free bowel content, pus, or blood	0.8	0	
Malignancy	No	18.5	11.9	0.390
	Localized tumor	40.3	45.9	
	Nodal metastasis	8.1	6.3	
	Distant metastasis	33.1	35.8	
Type of surgery	Elective	100	100	

* COPD: Chronic Obstructive Pulmonary Disease.

**Table 5:** Predictors of “textbook outcome” after multivariate logistic regression analysis.

		Univariate Analysis		Multivariable Analysis	
		Odds Ratio (95% CI)	*p* Value	Odds Ratio (IC al 95%)	*p* Value
Gender	Male Female	Ref 0.824 (0.55–1.34)	0.441		
Age (years)	≤60	Ref			
	61–70	0.66 (0.37–1.18)	0.158		
	≥70	0.67 (0.38–1.18)	0.165		
Cardiac signs	No failure	Ref			
	Diuretic, digoxin, antianginal or hypertensive therapy	1.03 (0.63–1.68)	0.917		
	Peripheral edema, warfarin therapy, borderline cardiomegaly	0.61 (0.24–1.59)	0.313		
Respiratory history	No dyspnea	Ref			
	Dyspnea on exertion, mild COPD on chest radiograph	0.82 (0.46–1.48)	0.512		
	Limiting dyspnea (1 flight) moderate COPD on chest radiograph	0.62 (0.18–2.08)	0.436		
Serum urea (mmol/L)	<7.5	Ref			
	7.5–10	0.73 (0.25–2.15)	0.572		
	10.1–15	0.42 (0.12–1.47)	0.174		
	>15	0.49 (0.08–2.98)	0.437		
Sodium (mmol/L)	≥136	Ref			
	131–135	0.76 (0.34–1.71)	0.517		
Potassium (mmol/L)	3.5–5	Ref			
	3.1–3.4 or 5.1–5.3	0.65 (0.21–1.99)	0.454		
Hemoglobin (g/dL)	13–16	Ref		Ref	
	11.5–12.9 or 16.1–17	0.67 (0.39–1.17)	0.158	0.68 (0.39–1.2)	0.185
	10–11.4 or 17.1–18	0.66 (0.31–1.41)	0.281	0.96 (0.43–2.24)	0.927
	<10 or >18	0.31 (0.11–0.87)	0.026	0.80 (0.23–2.75)	0.726
White cell count (×10^3^)	>4	Ref			
	10.1–20 or 3.1–3.9	0.79 (0.39–1.57)	0.504		
	>20 or <3.1	0.37 (0.03–4.11)	0.425		
Electrocardiogram	Normal	Ref			
	Atrial fibrillation (rate 60–90/min)	0.64 (0.23–1.83)	0.407		
	Any other abnormal rhythm, or ≥5 ectopic/min. Q waves or ST/T wave changes	0.15 (0.02–1.28)	0.08		
Operative severity	Major	Ref			

*p* Value in italics when results were statistically significant (<0.05).

## Data Availability

Data are disposable under request.

## Abbreviations

TO	Textbook Outcome
HPB	hepatopancreatobiliary
LOS	length of stay
POSSUM	Physiological and Operative Severity Score for the enUmeration of Mortality and morbidity
DP	distal pancreatectomy
PD	pancreatoduodenectomy
IQR	interquartile range
CHAID	chi-square automatic interaction detection
